# Evidence for the butyrate metabolism as key pathway improving ulcerative colitis in both pediatric and adult patients

**DOI:** 10.1080/21655979.2021.1985815

**Published:** 2021-10-21

**Authors:** Zheng Zhou, Jiasheng Cao, Xiaoming Liu, Mingsong Li

**Affiliations:** a Guangdong Provincial Key Laboratory of Gastroenterology, Department of Gastroenterology, Nanfang Hospital, Southern Medical University, Guangzhou, Guangdong Province, 510515, China; b Department of General Surgery, Sir Run-Run Shaw Hospital, Zhejiang University School of Medicine, Hangzhou, Zhejiang Province, 310016, China; c Department of Gastroenterology, Third Affiliated Hospital of Guangzhou Medical University, Guangzhou, Guangdong Province, 510000, China

**Keywords:** Butyrate, ulcerative colitis, pediatric, adult, bioinformatic

## Abstract

Accumulating evidence has shown many similarities and differences of gene profiles and pathways between pediatric and adult ulcerative colitis (UC) patients. In this study, we aimed to investigate the shared genes and pathways in intestinal tissues of pediatric and adult UC. Differentially expressed genes (DEGs) between pediatric and adult UC were identified *via* bioinformatic analysis of Gene Expression Omnibus datasets GSE87473 and GSE126124. Gene Ontology and pathway enrichment were used to analyze overlapped and distinguished DEGs. Gene Set Variation Analysis (GSVA) was utilized for contrast consistency. Mice colitis models were induced by dextran sulfate sodium (DSS) and *Citrobacter rodentium*. 2616 DEGs were screened out in intestinal tissues of adult UC compared with those of adult healthy controls, and 1195 DEGs in pediatrics. Same pathways between pediatric and adult UC were enriched using overlapped DEGs, mainly related to immune responses and metabolic processes, including butyrate metabolism, which was also identified by GSVA analysis. Of note, butyrate metabolism was the exclusive down-regulated pathway enriched by these two analyses, indicating that butyrate metabolism is one of the key pathways associated with both pediatric and adult UC. In addition, butyrate suppressed DSS-induced and *Citrobacter rodentium*-induced intestinal inflammation in mice. Therefore, the study revealed that butyrate metabolism was critical in both pediatric and adult UC. And butyrate suppressed colitis in mice, which provided a theoretical basis for the potential treatment of butyrate for UC patients.

**Abbreviations:** UC, Ulcerative colitis; IBD, Inflammatory bowel disease; DEGs, Differentially expressed genes; GEO, Gene Expression Omnibus; SVA, Spatial variant apodization; LIMMA, Linear models for the microarray data; FC, Fold change; GO, Gene Ontology; KEGG, Kyoto Encyclopedia of Genes and Genomes; GSVA, Gene Set Variation Analysis; MSigDB, Molecular Signatures Database; WT, Wild-type; DSS, Dextran sulfate sodium; HC, Healthy control; SD, Standard deviation; SNHG5, Small nucleolar RNA host gene 5; GLP-2, Glucagon-like peptide 2; GSE, Gene set enrichment; ECM, Extracellular matrix; TCA, Tricarboxylic acid cycle; NA, Not available.

## 1. Introduction

Ulcerative colitis (UC), which belongs to inflammatory bowel diseases (IBD), is a chronic inflammatory disease of the large intestine. Aberrant expression of various genes are associated with UC[1]. For example, abnormal gene expressions related to immune function are among the critical factors in the pathogenesis of UC [2]. Large quantities of cytokines are also involved in the occurrence and development of UC [3]. Moreover, apoptosis-related genes and cancer-related genes are closely related to intestinal lesions and carcinogenesis of UC [4]. There is no consensus that whether UC is caused by the infectious agents or genetic mutations. Indeed, UC does occur among family members, suggesting that genetic inheritance may be one of the causes [5]. No clinical or demographic parameters are typically associated with therapeutic evaluation, indicating that additional information is still required to improve the definition of patient diagnosis. Currently, the use of microbial markers or gene expression to support specific subsets of UC diagnosis and adjustment of treatment methods are limited.

Diet involvement plays an important role in the pathogenesis and management of UC both in pediatric and adult patients [6,7]. The typical Western diet (high sugar intake with low intake of vegetables) may contribute to the development of UC [8], because intestinal inflammation can be influenced in several pathways, including affecting the intestinal permeability, altering the gut microbiome, and dietary constituents acting as food antigens [9]. As for the role of diet in the management of UC, enteral nutrition, which is supplemented with colostrum, probiotics, and transforming growth factor-β, produces limited treatment responses in UC patients [10]. However, short-chain fatty acids, which are primary products of dietary fiber, consisting of acetate, propionate, and butyrate, could maintain intestinal homeostasis by increasing IL-22 in CD4 + T cells and innate lymphoid cells [11], so that it could improve clinical outcomes potentially.

Accumulating evidence demonstrated that there are similarities and differences between pediatric and adult UC in pathologic changes, symptoms, extraintestinal manifestations, and disease severity. For example, UC tends to be more extensive and aggressive in pediatric UC patients than adult counterparts, and monogenic etiologies are more highly represented in pediatric-onset patients compared with those diagnosed at an older age [12]. Various transcriptomic studies were performed to uncover the gene profiles of UC, including several efforts to discriminate between disease phases [13–15], sample sources (blood versus tissue) [16], microbiota association [17], genders [18], etc. However, the similarity and difference of gene profiles between pediatric and adult UC are still not completely characterized.

In this study, we aimed to identify the overlapped differentially expressed genes (DEGs) between pediatric and adult UC, and then the DEGs were enriched in several essential pathways and functions. We hypothesized that butyrate metabolism would be critical in both pediatric and adult UC patients, which would provide a theoretical basis for the potential treatment of butyrate for UC patients.

## 2. Materials and methods

### 2.1. Datasets and processing

GSE87473^19^ and GSE126124^20^ were retrieved from Gene Expression Omnibus (GEO) database (http://www.ncbi.nlm.nih.gov/geo/) [19], and the expression profiling arrays were generated using GPL13158^19^ and GPL6244^20^, respectively. All samples in those microarrays were divided into four groups based on age and disease status: pediatric control, pediatric UC, adult control, and adult UC (Supplementry Material 1). To remove the batch effects and normalization, the algorithm of spatial variant apodization (SVA) was performed [20]. After that, the *k*-nearest neighbors [21] algorithm was used to process the missing value. Averages were considered gene expression values if the multiple probes were mapped into the same gene symbol.

### 2.2. DEGs in intestine tissues

To analyze the DEGs in adult control (n = 21) and UC (n = 87), pediatric control (n = 20) and UC (n = 39), linear models for the microarray data (LIMMA) package were used to analyze gene profiles of tissue samples in R Studio (Version 3.6.3). Benjamini-Hochberg method was used to control the false discovery rate [22]. Gene expression values with log_2_ fold change (FC) > 0.5 and adjusted *p*-value < 0.05 were defined as the cutoff criteria. Hierarchical clustering analysis was used to build a hierarchy of clusters of the DEGs [23]. After getting the DEGs of tissues in pediatrics and adults, we took the intersection of those groups using VennDiagram (http://www.ehbio.com/Cloud_Platform/front). Overlapped and distinguished DEGs in pediatrics and adults were selected.

### 2.3. Function and pathway enrichment

These two analyses were based on the Metascape online database (http://metascape.org/) [24]. After inputting the corresponding shared genes from 2.2, genes were collected and grouped into clusters based on the membership similarities. And the parameters were set as follows: (1) p-values < 0.01, (2) minimum counts of 3, and (3) enrichment factors > 1.5. The functional molecular clusters of Gene Ontology (GO) and pathway clusters Kyoto Encyclopedia of Genes and Genomes (KEGG) were selected as functional pathways. More specifically, *p*-values were calculated based on the accumulative hypergeometric [25], and *q*-values using the algorithm of Benjamini-Hochberg were used to account for those comparisons [26]. Moreover, to perform hierarchical clustering on the enriched terms, Kappa scores [27] were also used as similarity metrics.

### 2.4. Gene Set Variation Analysis (GSVA)

GSVA is a nonparametric and unsupervised method for estimating changes in gene set enrichment through samples of expression data sets [28]. The pathway datasets of GO and KEGG were downloaded from Molecular Signatures Database (MSigDB) v7.2 (https://www.gsea-msigdb.org/gsea/msigdb/index.jsp) [29], and tissue probe matrix was used to perform the pathway matrix both in pediatrics and adults. In the study, GSVA was applied to build pathway-centric models of biology and to explore the potential regulatory pathways. The results of 2.3 and 2.4 were combined to identify the significant pathways in pediatrics and adults.

### 2.5. Mice

Specific pathogen-free C57BL/6 wild-type (WT) mice were purchased from Guangdong Medical Laboratory Animal Center. Mice were housed under standard conditions and cared for according to the institutional guidelines for animal care. The protocols for animal experiments followed the international guidelines and were approved by the Animal Care Committee of the Southern Medical University of China.

### 2.6. Citrobacter rodentium infection model

WT mice were orally infected with *Citrobacter rodentium* (5 × 10^8^/ mouse), and mice were administered with or without 200 mM butyrate in drinking water. Mice were sacrificed on day 10. Mouse weights were monitored daily.

### 2.7. Dextran sulfate sodium (DSS)-induced colitis model

WT mice were administered with 2% DSS in the presence or absence of 200 mM butyrate in drinking water for 7 days, followed by another 3 days of water with or without 200 mM butyrate. Mice were sacrificed on day 10. Mouse weights were monitored daily.

### 2.8. The inflamed colons of adult UC patients

All patients with UC were recruited from the Department of Gastroenterology, the Third Affiliated Hospital of Guangzhou Medical University. The diagnosis of UC was based on clinical symptoms, endoscopic examination and histological findings. The Institutional Review Board approved this study for Clinical Research of the Third Affiliated Hospital of Guangzhou Medical University (2,021,063). And the characteristics of healthy controls (HC) and UC patients were described in Supplementary Material 2.

### 2.9. Quantitative RT-PCR

The total RNA was extracted from the colon with TRIzol and followed by cDNA synthesis. Quantitative PCRs were performed by using SYBR Green Gene Expression Assays for *EHHADH, HMGCS2, ACSM3*, and *BDH2* on a Bio-Rad iCycler (Bio-Rad Laboratories, Hercules, CA), and all data were normalized to *GAPDH* mRNA expression. All the primers were ordered from Integrated DNA Technologies, and listed in Supplementary Material 3.

### 2.10. Histopathological assessment

At necropsy, mice colons were separated, and Swiss rolls of each were prepared. Tissues were fixed in 10% buffered formalin. Following paraffin embedding, 5-μm sections were prepared and stained with H&E. The severity of colitis was evaluated by a pathological scoring system [30]. The score of 0–3, denoting increasingly severe abnormality, was assigned for each of these parameters and added together to calculate the overall histological score (no signs of inflammation, 0; increased mucosal mononuclear cells were present, 1; increased mucosal and submucosal or transmural mononuclear cells were present, 2; mucosal regeneration with crypt distortion as well as increased crypt proliferation or erosions or ulcers, 3).

### 2.11. Statistical analysis

Student’s *t*-test was used for comparison when data were normally distributed, and Mann–Whitney *U* test was used to measure the difference when data were not normally distributed. All the tests were two-sided. All the data were analyzed using Prism 9.0 (GraphPad Software, San Diego, CA), and presented as mean ± standard deviation (SD). All the data were presented as mean ± SD. *p < 0.05, **p < 0.01, ***p < 0.001.

### 2.12. Ethical considerations

The research was carried out according to the Helsinki Declaration and approved by the Ethics Committee of Nan Fang Hospital, Southern Medical University, Guangzhou, Guangdong Province, China (No. L2017010).

## 3. Results

In this bioinformatics study, we found that gene expressions in UC differed from healthy controls both in pediatric and adult populations. The overlapped DEGs between pediatric and adult UC were identified and then they were enriched in several essential pathways and functions. Notably, butyrate metabolism was critical in both pediatric and adult UC patients. And butyrate suppressed colitis in mice, potentially supporting the treatment of butyrate for UC patients.

### 3.1. Study population and design

**Figure 1.**
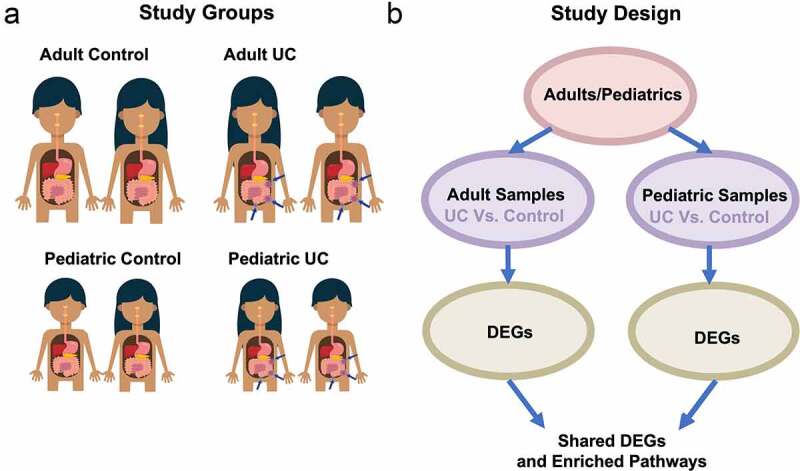
Study population and design. (a) Study groups were divided into pediatric and adult groups, and each group includes healthy controls and patients with ulcerative colitis. (b) Flow diagrams summarize the study process and enrichment analyses

In GSE87473 dataset [31], 19 colon samples of pediatric UC patients, 87 colon samples of adult UC patients, and 21 colon samples of adult controls with specific age and disease extent were reported. Meanwhile, in GSE126124 dataset [32], 21 colon tissues of pediatric controls and 20 colon tissues of pediatric UC patients with specific age and gender were reported. After a combination of GSE87473 and GSE126124 datasets (Supplementary Material 1), four groups (pediatric UC, pediatric control, adult UC, and adult control) with intrinsic details of age, including the pediatric group (6–18 years old) and the adult group (>18 years old), were included in the further analysis (Figure 1(a)). Gene expressions of colon biopsies from adult UC patients and healthy controls, as well as pediatric UC patients and healthy controls, were analyzed to identify molecular signatures of UC in pediatrics and adults. These analyses elucidated key genetic functions and pathways involved in disease pathogenesis, which can be used to identify the shared DEGs, functions, and pathways in pediatric and adult UC patients (Figure 1(b)).

### 3.2. Functional enrichment analyses of overlapped DEGs in intestinal tissues of pediatric and adult UC

**Figure 2.**
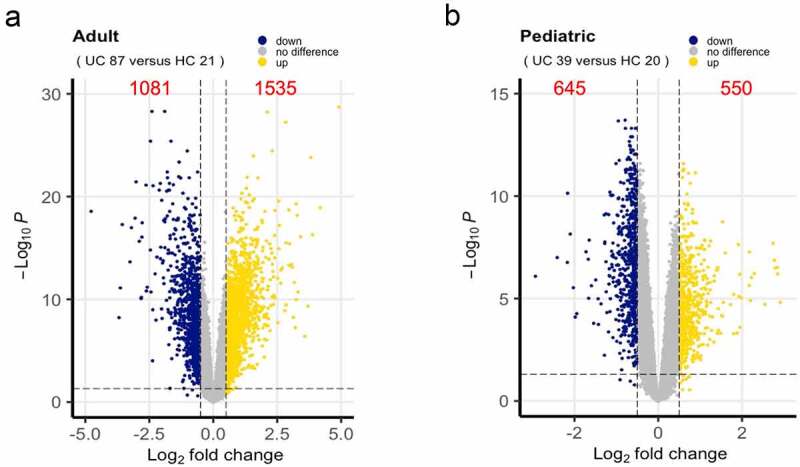
DEGs between UC and healthy control. (a) DEGs in adults in Volcano plots. (b) DEGs in pediatrics in Volcano plots. Each symbol represents a gene, and gold or blue color indicates up-regulated or down-regulated genes, respectively. DEGs, Differentially expressed genes; UC, Ulcerative colitis

After screening DEGs based on the cutoff criteria of *p*-value < 0.05 and [log_2_FC] > 0.5 (Supplementary Material 4), 3811 DEGs in intestinal tissues of pediatric and adult UC were shown in volcano plots (Figure 2). A total of 2616 DEGs, including 1535 up-regulated genes and 1081 down-regulated genes, were screened out in intestinal tissues of adult UC compared with those of healthy adult controls (Figure 2(a)), while 1195 DEGs, including 550 up-regulated genes and 645 down-regulated genes, were found in intestinal tissues of pediatric UC compared with those of healthy pediatric controls (Figure 2(b)).

**Figure 3.**
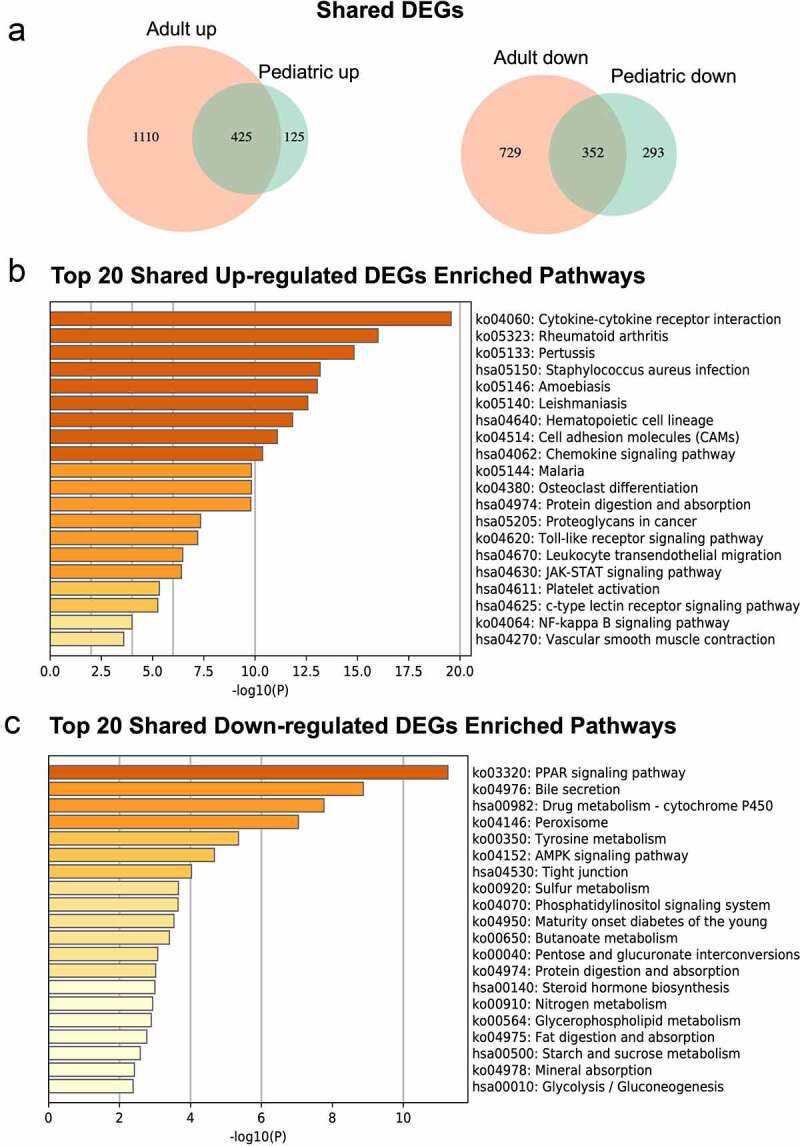
Enrichment analyses of overlapped DEGs in pediatric and adult UC. (a) Overlapped DEGs in pediatric and adult UC were shown in Venn diagram. (b) Top 20 KEGG pathways were enriched using overlapped up-regulated DEGs. (c) Top 20 KEGG pathways were enriched using overlappped down-regulated DEGs. The enriched analyses were ranked by p-value. DEGs, Differentially expressed genes; UC, Ulcerative colitis; KEGG, Kyoto Encyclopedia of Genes and Genomes

To further identify similar and distinguished DEGs in intestinal tissues between pediatric and adult UC, we compared these two sets of UC patients’ data. 425 up-regulated genes and 352 down-regulated genes were overlapped in pediatric and adult UC (Figure 3(a)). As shown in Figure 3b, overlapped up-regulated DEGs were mainly enriched in pathways, such as cytokine-cytokine receptor interaction, rheumatoid arthritis, cell adhesion molecules, pertussis, and staphylococcus aureus infection, while function analysis revealed that GO sets such as extracellular matrix structural constituent, glycosaminoglycan binding, integrin binding, cytokine activity, carbohydrate binding and so on were significantly associated with the overlapped up-regulated genes between pediatric and adult UC, of which top 20 GO sets were shown in Supplementary Material 5A. Meanwhile, the top 20 enriched pathways and GO sets of overlapped down-regulated genes were shown in Figure 3(c) and Supplementary Material 5B. Interestingly, metabolism was the most relevant biological process when analyzing similar down-regulated DEGs in pediatric and adult UC. Seven different metabolic processes were enriched, including drug metabolism-cytochrome P450, tyrosine metabolism, sulfur metabolism, butyrate metabolism, nitrogen metabolism, glycerophospholipid metabolism, and starch and sucrose metabolism.

### 3.3. GSVA analysis of tissue microarray data

**Figure 4.**
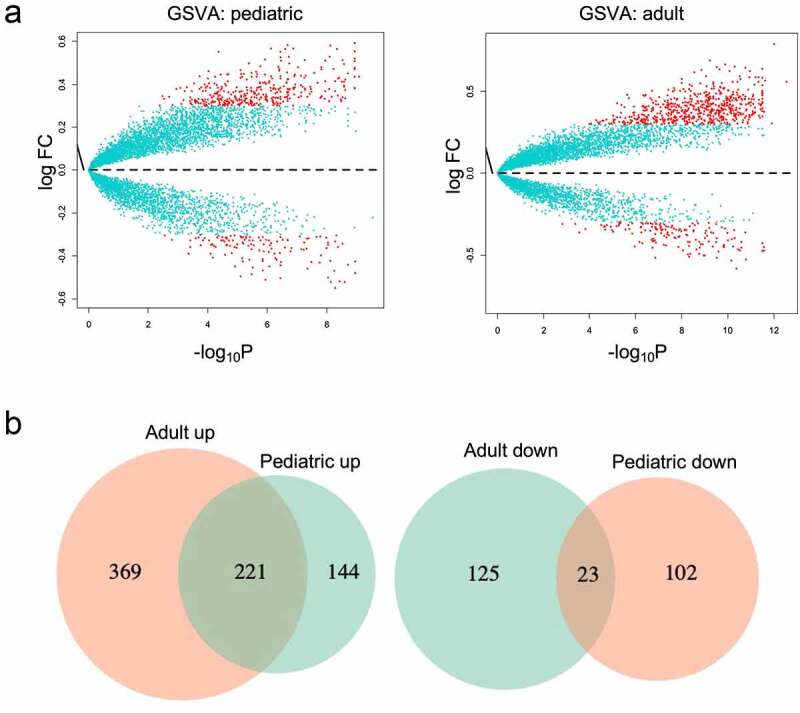
Shared gene sets between pediatric and adult UC using GSVA. (a) The differences in pathway enrichment between UC and healthy control in pediatric and adult were visualized by Volcano plot using GSVA. (b) Venn diagram of shared enrichment pathways in pediatric and adult UC. UC, Ulcerative colitis; GSVA, Gene Set Variation Analysis

A non-parametric unsupervised method GSVA was accessed for gene set enrichment (GSE) in colonic tissue microarray data. It analyzed the changes in the coordinate system and converted the data from a sample matrix gene to a sample matrix set gene, then the enrichment of each sample could be evaluated [33]. This transformation is completed without using a phenotype, thus facilitating compelling and open analysis based on the current path. As shown in Figures 4(a), 590 and 148 enrichment analyses were associated with up-regulated genes and down-regulated genes were found in adult UC compared with healthy adult controls, respectively, while 365 and 125 enrichment analyses associated with up-regulated genes and down-regulated genes were found in pediatrics. We also found that there were 221 and 23 enrichment analyses associated with up-regulated pathways and down-regulated pathways in both pediatric and adult UC (Figure 4(b)). And KEGG associated pathways were shown in Table 1.

**Table 1.** Shared KEGG pathways enrichment analysis of GSVA

Description	LogFC	AveExpr	Adj.P.Val	LogFC		AveExpr	Adj.P.Val
	Pediatric				Adult		
**Shared up-regulated pathways**							
Allograft rejection	0.439	0.019	0.000188529	0.636		0.023	2.38E-10
Autoimmune thyroid disease	0.339	0.010	0.000393567	0.540		0.025	7.11E-12
Cell adhesion molecules cams	0.344	0.006	1.23E-05	0.470		0.010	1.75E-11
Complement and coagulation cascades	0.378	0.015	5.36E-07	0.391		0.009	7.83E-11
Cytokine-cytokine receptor interaction	0.325	0.003	5.96E-06	0.426		0.016	4.53E-12
ECM receptor interaction	0.438	0.007	1.25E-07	0.417		0.014	4.26E-09
Glycosaminoglycan biosynthesis chondroitin sulfate	0.497	0.001	1.87E-07	0.469		0.027	8.60E-12
Glycosaminoglycan degradation	0.478	0.018	1.54E-08	0.347		0.010	1.20E-08
Glycosphingolipid biosynthesis ganglio series	0.425	0.004	2.01E-07	0.418		0.007	4.10E-06
Graft versus host disease	0.461	0.024	9.72E-05	0.647		0.032	3.00E-10
Intestinal immune network for IgA production	0.368	0.006	0.000345337	0.533		0.009	4.10E-10
Leishmania infection	0.397	0.007	4.99E-05	0.521		0.016	1.41E-10
Proteasome	0.332	0.029	0.00296787	0.502		0.034	7.09E-09
Protein export	0.341	−0.001	0.00196511	0.471		0.022	4.20E-07
Systemic lupus erythematosus	0.368	0.022	0.000148244	0.517		0.017	4.10E-10
Type I diabetes mellitus	0.382	0.023	0.000116374	0.587		0.021	1.18E-11
Viral myocarditis	0.328	0.008	1.13E-05	0.376		0.014	7.10E-09
**Shared down-regulated pathways**							
Aminoacyl tRNA biosynthesis	−0.324	0.013	0.001582178	−0.340		−0.005	0.000154852
Butanoate metabolism	−0.324	0.013	0.001582178	−0.581		−0.008	4.30E-11
Citrate cycle TCA cycle	−0.495	0.013	7.95E-06	−0.514		0.001	1.40E-07

KEGG, Kyoto Encyclopedia of Genes and Genomes; GSVA, Gene Set Variation Analysis; ECM, Extracellular matrix; TCA, Tricarboxylic acid cycle.

### 3.4. Identify butyrate metabolism as a key pathway associated with UC in both pediatric and adult populations

Next, we compared the results which were obtained from the above two analyses, and we found that 8 pathways (7 up-regulated and 1 down-regulated) were presented in these two analyses (Table 2). Of note, butanoate metabolism, which was also called butyrate metabolism, was the only one down-regulated pathway, indicating butyrate metabolism may be a key pathway associated with UC in both pediatric and adult populations.

**Table 2.** Comparison of GSVA and metascape analysis

Description	LogP	Symbols
**Shared up-regulated pathways**		
Cytokine activity	−14.46	FGF2, CXCL1, CXCL2, CXCL3, IFNG, IL1A, IL1B, IL1RN, IL6, INHBA, CXCL10, MIF, CXCL9, TNFRSF11B, OSM, CCL2, CCL11, CCL18, CXCL6, CXCL11, CXCL5, SPP1, TIMP1, WNT5A, TNFSF13, GREM1
Complement and coagulation cascades	−12.41	CFB, C1R, C1S, C2, C4BPA, C4BPB, CD55, F3, CFI, CXCL10, ITGB2, SERPINE1, SERPINA1, PLAU, PLAUR, VWF
Cytokine-cytokine receptor interaction	−18.79	TNFRSF17, CSF2RB, CSF3R, CXCL1, CXCL2, CXCL3, IFNG, IL1A, IL1B, IL1RN, IL2RA, IL3RA, IL6, CXCR1, CXCR2, TNFRSF9, INHBA, CXCL10, KDR, CXCL9, TNFRSF11B, OSM, PDGFRB, CCL2, CCL11, CCL18, CXCL6, CXCL11, CXCL5, TIMP1, TNFRSF1B, TNFSF13, OSMR, IL21 R, TNFRSF12A
ECM receptor interaction	−9.22	COL1A1, COL1A2, COL4A1, COL4A2, COL6A2, COL6A3, FN1, TNC, ITGA5, LAMC1, LAMC2, SPP1, VWF
Graft versus host disease	−4.18	HLA-DMA, HLA-DRB1, IFNG, IL1A, IL1B, IL6
Systemic lupus erythematosus	−3.39	C1R, C1S, C2, FCGR1A, FCGR2A, HLA-DMA, HLA-DRB1, IFNG, CXCL10
Type I diabetes mellitus	−3.15	HLA-DMA, HLA-DRB1, IFNG, IL1A, IL1B
**Shared down-regulated pathways**		
Butanoate metabolism	−3.29	EHHADH, HMGCS2, ACSM3, BDH2

GSVA, Gene Set Variation Analysis; ECM, Extracellular matrix.

### 3.5. The validation of gene expression levels in adult UC patients

**Figure 5.**
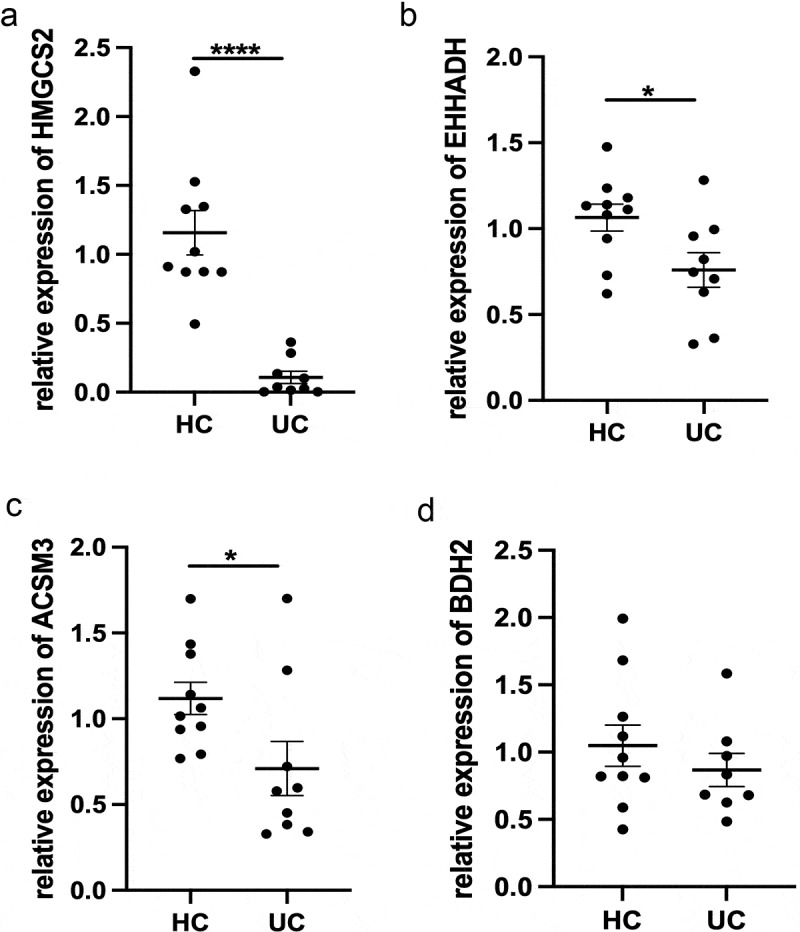
Expression of (a) *HMGCS2*, (b) *EHHADH*, (c) *ACSM3*, and (d) *BDH2* mRNA in colonic mucosa of healthy patients and inflamed colonic mucosa of UC patients. Gene expression was normalized to *GAPDH* in each sample. *p < 0.05, ****p < 0.0001. UC, Ulcerative colitis

Based on our previous analysis, we found four genes showed downregulation associated with butyrate metabolism, and we validated colonic *HMGCS2, EHHADH, ACSM3*, and *BDH2* expression in the inflamed mucosa of UC patients. As shown in Figure 5(a-c), *HMGCS2, EHHADH*, and *ACSM3* were significantly decreased in the inflamed mucosa of UC patients compared with healthy controls. However, the expression of *BDH2* was almost at the same level both in UC patients and healthy controls (Figure 5(d)).

### 3.6. The protective role of butyrate in regulation of intestinal inflammation in mice

**Figure 6.**
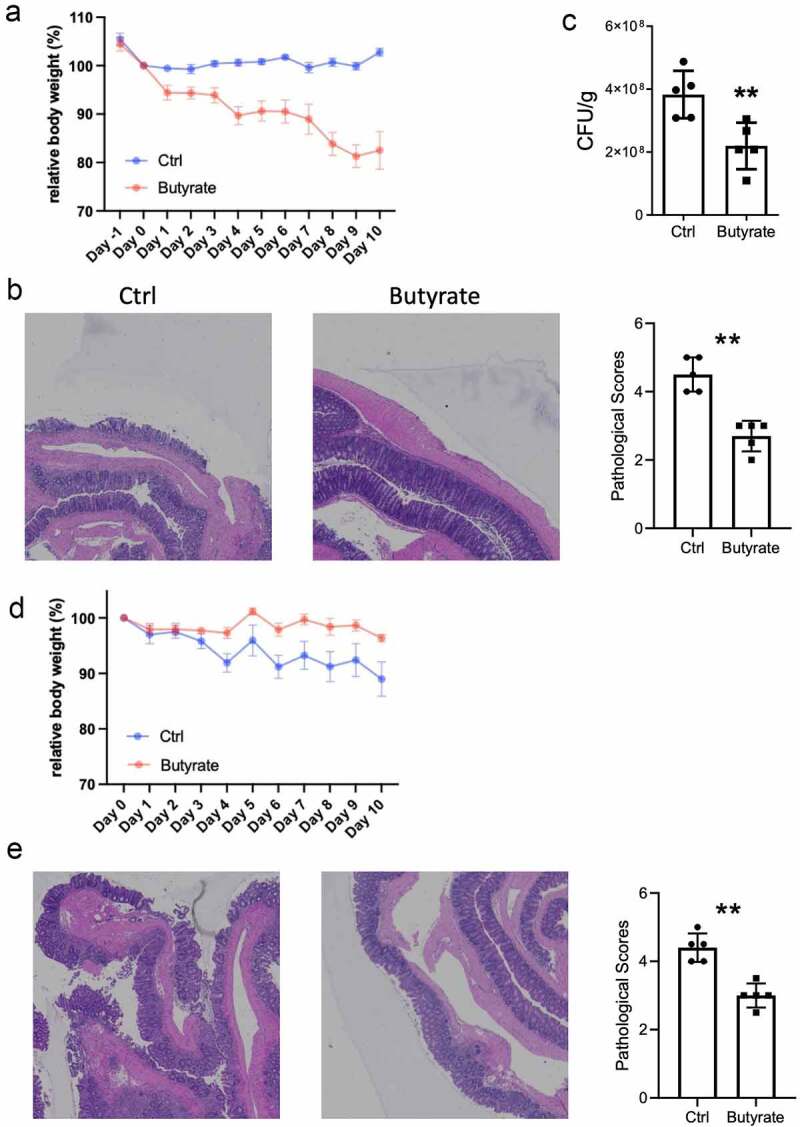
Butyrate inhibits intestinal inflammation in mice. (a) Mice were orally treated with *C.rodentium* (5 × 10^8^ CFU/mice) and treated with or without butyrate (200 mM) in drinking water for 10 days. Monitored mice weight every day. (b) Colonic histopathology was analyzed when mice were sacrificed on day 10. (c) Collected the feces and measured the CFU on day 4. (d) Mice were administrated with 2% DSS in drinking water for 7 days and switched to untreated water for another 3 days. Treated with or without butyrate (200 mM) in drinking water for 10 days, and mouse weights were monitored daily. (e) Histopathological change and histological score in of colon in mice colitis models. **p < 0.01. CFU, Colony forming unit; DSS, Dextran sulfate sodium

**Figure 7.**
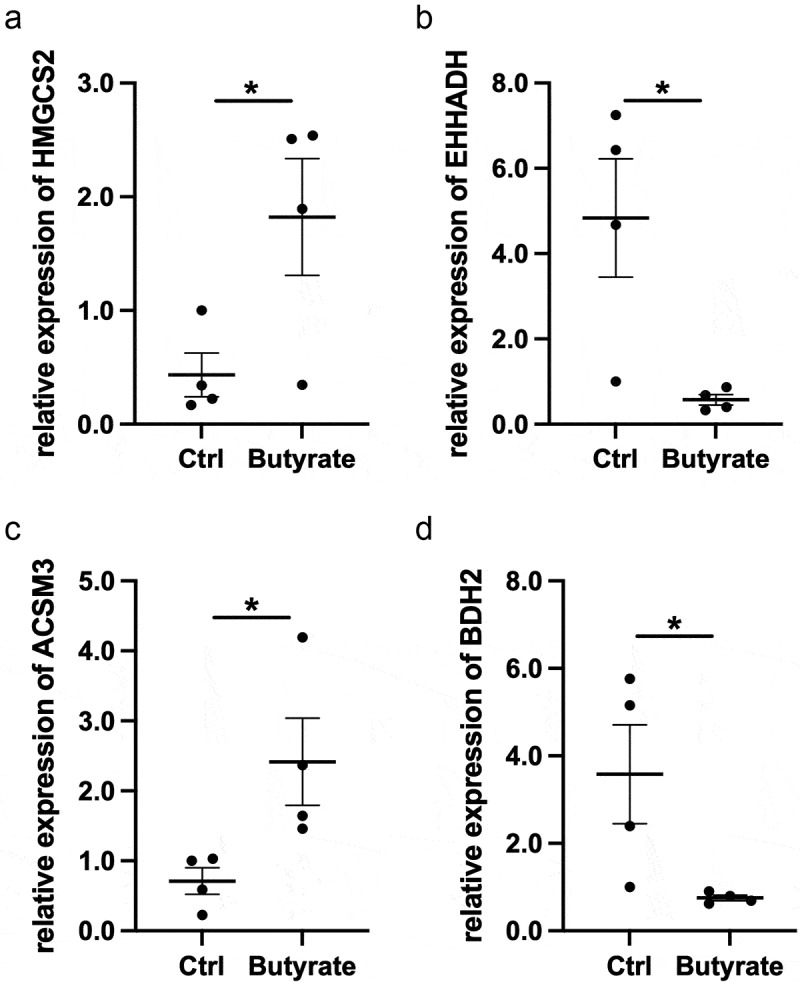
Expression of (a) *HMGCS2*, (b) *EHHADH*, (c) *ACSM3*, and (d) *BDH2* mRNA in DSS-induced mice with or without butyrate. Gene expression was normalized to *GAPDH* in each sample. *p < 0.05. DSS, Dextran sulfate sodium

Given that butyrate is closely associated with UC, we next investigated the role of butyrate in regulation of colitis. We induced colitis using *Citrobacter rodentium* infection models. Mice were infected with *Citrobacter rodentium* and administered with or without butyrate in drinking water. We found that feeding butyrate prevented mice against weight loss and suppressed intestinal inflammation in mice infected with *Citrobacter rodentium* (Figure 6(a,b)). Mice fecal C. *rodentium* CFU alos decreased after administrated with butyrate (Figure 6(c)). To further confirm the protective role of butyrate in suppressing colitis, we induced colitis using DSS in mice, and mice were given with or without butyrate in drinking water. Similar to *Citrobacter rodentium* infection model, butyrate suppressed intestinal inflammation, which was evidenced by decreased weight loss and lower pathology scores (Figure 6(d,e)). Moreover, we identified the genes expression regulations after butyrate use in mice models with intestinal inflammation using RT-PCR. The expression of *HMGCS2* and *ACSM3* were significantly higher in the group that combined butyrate with DSS-induced than those only treated with DSS, while the expression of *EHHADH* and *BDH2* were significantly decreased in butyrate-treated groups (Figure 7). Taken together, these results demonstrated that butyrate protected the intestines from inflammation induced by both intestinal acute injury and enteric infection.

## 4. Discussion

Currently, the diagnosis of UC mainly depends on epidemiological data, clinical performance, and systematic examination, including laboratory examination, digestive endoscopy, imaging, and pathological examination. However, the diagnosis of UC, including pediatric and adult UC, still lacks gold standards. Therefore, it is necessary to understand the mechanisms underlying the pathogenesis of UC better, and it is crucial to identify the similarities and differences of gene expressions and pathways between pediatric and adult UC. In this bioinformatics study, based on GSE87473^19^ and GSE126124^20^ datasets, four groups (pediatric UC, pediatric control, adult UC, and adult control) with intrinsic details of age were included in the analysis, and we found that gene expressions in UC differed from healthy controls both in pediatric and adult populations. In addition, overlapped DEGs between pediatric and adult UC were enriched in several essential pathways and functions, in which butyrate metabolism was critical in both pediatric and adult UC populations.

Interestingly, based on GSVA, we found that butanoate metabolism was the only one down-regulated pathway after two analyses, indicating a critical role for butanoate metabolism in regulating the pathogenesis of UC in both pediatrics and adults. Moreover, butyrate is a cooperative product of many symbiotic bacteria in the intestine. Many studies have confirmed that there was a variety of symbiotic bacteria in the intestine that can produce butyrate directly [34–36]. A small number of bacteria promote the growth of specific bacteria to increase the production of butyrate. Synthetic short-chain fatty acid precursors indirectly lead to an increase in butyrate content [37]. Therefore, supplementation of butyrate can correct the intestinal bacterial dystrophy caused by UC and adjust the excessive release of pro-inflammatory cytokines by activating multiple metabolism-related pathways. Moreover, butyrate activates the regulatory of T cells, further reducing the production of TNF-α and IL-6 [38,39], meanwhile, it could increase the expression of Hif1α and Ahr in CD4 T cells to promote the production of cytokine IL-22, leading to inhibiting intestinal inflammation [11]. In addition, butyrate was also shown to have the ability to inhibit a variety of pro-inflammatory signaling pathways through histone deacetylase inhibitor-dependent and independent mechanisms [40].

It is contradictory that several studies suggested that butyrate supplementation could be beneficial for UC patients [41,42], while other studies showed no effect or even negative effect on intestinal inflammation [43,44]. Specifically, butyrate caused more significant down-regulation on gene expression of inflammatory pathways in colon tissues without inflammation of UC patients that those with inflammation [45]. Meanwhile, Vancamelbeke et al [46]. concluded that butyrate supplementation could not protect against inflammation-induced negative effects on epithelial barrier function. Instead, it strongly up-regulated the expression of inflammatory mRNA and protein. The above-mentioned could partly explain why anti-inflammatory effects of butyrate in UC patients are not always acquired, and the mechanisms of butyrate on UC should be investigated in future studies. However, due to the small sample sizes of both two studies, the results should be further validated. Despite of the indecisive results, butyrate is considered as a therapeutic alternative for UC patients based on its potential anti-inflammatory properties.

It is critical to compare our study with the previous studies on the investigation of butyrate metabolism. For example, the study performed by Preter et al [47]. concluded that the deficiency of butyrate metabolism in UC patients was caused by the decreased expression level of *SLC16A1* and enzymes in the butyrate oxidation pathway. And there were differences between our study and the above-mentioned. First, both pediatric and adult UC patients and controls were included in our study instead of merely including adult populations, which may improve the generality of our results. Second, our study newly added *EHHADH, HMGCS2, BDH2*, and RT-PCR further validated the decreased expression of those three genes in UC patients. Third, due to the deficiency of details of treatment in GSE87473 and GSE126124 datasets, the treatment response of gene expression was not investigated in our study. Meanwhile, the results of our study were not further validated in pediatric UC patients owing to the lack of pediatrics in our hospital. And a further multi-center study with complete details of both pediatric and adult UC patients would be carried out to investigate the gene expression after treatment and make a further validation of the results.

Several characteristics of the patients, including gender, country, and treatment, may affect the butyrate metabolism pathway. There are limited studies being performed to investigate whether gender, and country affect the butyrate metabolism pathway. Indeed, gender and country factors are associated with the risk of UC development (accompanied by impaired butyrate metabolism) [48]. On the one hand, as for gender factor, a higher prevalence of smoking in males might have caused the higher mortality rate of UC than females, because smoking is considered one of the most significant environmental factors of UC [49]. And the influence of sex hormones on UC prognosis based on the brain-gut–microbiota axis is still not completely studied. On the other hand, as for country factor, the country development degree and the related genetic factors contribute to the UC development. The incidence of UC is significantly lower in developing countries than in developed ones [48]. And the genetic risk factors are different between Asian and Western regions in UC [50]. Furthermore, the treatment factor may affect the butyrate metabolism pathway. Preter et al [47]. found that the originally decreased gene expression of *ACSM3* in butyrate metabolism pathway could return to control values after infliximab therapy.

There were eight pathways found in the study, including seven shared up-regulated pathways and one shared down-regulated pathway (butyrate metabolism). As for the butyrate metabolism, the connection between these four genes, including (*ACSM3, EHHADH, HMGCS2*, and *BDH2*) and butyrate metabolism pathways should be illustrated (Supplementary Material 6). First, *ACSM3* participates in the synthesis of butyryl-CoA. Next, *EHHADH* is associated with the synthesis of Acetoacetyl-CoA which then creates 3-Hydroxymethyl-glutaryl-CoA participated with *HMGCS2*. And *BDH2* joins the acetoacetate to create 3-Hydroxybutanoate. Currently, biologic treatment for UC mostly target symbols such as TNF and IL in up-regulated pathways, leading to more researches focusing on inhibiting symbols in up-regulated pathways. For example, the inhibition of IL1B and IL6 could ameliorate colitis based on the anti-inflammatory and anti-oxidant activity [51], both of which were involved in the shared up-regulated pathways, including cytokine activity, cytokine-cytokine receptor interaction, graft versus host disease, and type I diabetes mellitus. Meanwhile, CXCL10 was associated with UC progression[1], and it was up-regulated in cytokine activity, complement and coagulation cascades, cytokine-cytokine receptor interaction, and systemic lupus erythematosus, which shed light on the drug target for UC patients. After a comparison of pediatric and adult UC patients, the shared pathways would be identified to improve the generality of the study. Considering the overlaps of symbols in the shared up-regulated pathways, researchers could focus on targeting the overlapped symbols to inhibit the up-regulated pathways to suppress colitis.

Besides the butyrate supplementation as a potential treatment for UC patients, several emerging alternatives could be effective in the treatment of UC. In the aspect of gene, the knockdown of the lncRNA (small nucleolar RNA host gene 5) SNHG5 could counteract TNF-α reduced dysfunction of intestinal epithelial cells in UC patients, which may be a promising therapy for UC [52]. Meanwhile, in the aspect of protein, increased serum glucagon-like peptide 2 (GLP-2) level, a hormone secreted by L-cells of intestinal mucosa, inducing reduced microbiota diversity and abundance, may be considered a treatment for UC patients [53].

However, there are still some limitations to be addressed. First, the sample size was small without gender, country, and treatment factors. And an improved bioinformatic analysis with a large sample size would be conducted to improve the accuracy of association between genes and UC both in pediatric and adult patients. Additionally, the four genes enriched in the butyrate metabolism were not measured and validated in pediatric UC patients due to the lack of pediatric patients in our hospital. And a further multi-center study with both pediatric and adult UC patients would be carried out to validate the hypothesis. Finally, the molecular network and genes involved in UC would be investigated to better dissect how to counteract UC onset and progression, such as deeply studying samples from UC patients.

## 5. Conclusion

The study revealed that butyrate metabolism was critical in association with both pediatric and adult UC. And butyrate suppressed colitis in mice, which provided a theoretical basis for the potential treatment of butyrate for UC patients.

## Supplementary Material

Supplemental Material

## Data Availability

The datasets used and/or analyzed during this study are available from the corresponding author upon reasonable request.

## Research highlights

- Shared genes and pathways in intestinal tissues of pediatric and adult UC were explored.
- Butyrate metabolism was critical in association with both pediatric and adult UC.
- Butyrate suppressed colitis in mice models.
- The study provided a theoretical basis for the potential treatment of butyrate for UC patients.
