# Supplementary material for: Evidence for the butyrate metabolism as key pathway improving ulcerative colitis in both pediatric and adult patients
Source: Bioengineered. 2021 Oct 21;12(1):8309–24. doi: 10.1080/21655979.2021.1985815 (PMC8806981; doi:10.1080/21655979.2021.1985815)
Supplement: Supplemental Material [file KBIE_A_1985815_SM9367.zip › supplementary/Supplementary Material 2.docx]

| **Supplementary Material 2**: Clinical characteristics of patients with HC and UC. | | |
| --- | --- | --- |
|  | HC (n = 10) | UC (n = 9) |
| Age (y) | 47.20 ± 2.13 | 45.22 ± 4.51 |
| Gender |  |  |
| Male | 3 | 5 |
| Female | 7 | 4 |
| Current therapy |  |  |
| 5-aminosalicylates | NA | 5 |
| Immunosuppressants | NA | 2 |
| Biologics | NA | 1 |
| Nutritional therapy | NA | NA |
| Disease location^#^ |  |  |
| E1 | NA | 4 |
| E2 | NA | 4 |
| E3 | NA | 1 |

#According to the Montreal classification system. HC, Healthy control; UC, Ulcerative colitis; NA, Not available.
