# Supplementary material for: Evidence for the butyrate metabolism as key pathway improving ulcerative colitis in both pediatric and adult patients
Source: Bioengineered. 2021 Oct 21;12(1):8309–24. doi: 10.1080/21655979.2021.1985815 (PMC8806981; doi:10.1080/21655979.2021.1985815)
Supplement: Supplemental Material [file KBIE_A_1985815_SM9367.zip › supplementary/Supplementary Material 3.docx]

**Supplementary Material 3**: Primers for human and mice qRT-PCR.

| Genes | Species | Forward Primer | Reverse Primer |
| --- | --- | --- | --- |
| *Gadph* | Human | GTCTCCTCTGACTTCAACAGCG | ACCACCCTGTTGCTGTAGCCAA |
| *Acsm3* | Human | GATACGGGCTGGGCAAAGTCTG | TCGGCTCAAAACGGGGTAAATGG |
| *Hmgcs2* | Human | AGGCTGGAAGTAGGCACTGAGAC | CCGTAGCAGGCATTGGTGGTATC |
| *Ehhadh* | Human | GAAGAAGGCAGCAAACCAGAGGAG | CCAACCCAGCAAGATCAGACACTC |
| *Bdh2* | Human | CCAGGAACAGTTGATACGCCATCTC | CAGTTGCGAATCTTCCCGTCTTTTG |
| *Gadph* | Mouse | AACTTTGGCATTGTGGAAGG | ACACATTGGGGGTAGGAACA |
| *Acsm3* | Mouse | ACTCCTCCGTCAAACTCCGT | TTGGCCAAGTCCATTGCCTCT |
| *Hmgcs2* | Mouse | AGCTACTGGGATGGTCGCTA | ACGCGTTCTCCATGTGAGTT |
| *Ehhadh* | Mouse | CGGTCAATGCCATCAGTCCA | AGCACCTGCACAGAAGTTGT |
| *Bdh2* | Mouse | GATCGGTCTCACCAAGTCCG | GTGTCAACCGTTCCTGGACA |
