# Supplementary material for: Evidence for the butyrate metabolism as key pathway improving ulcerative colitis in both pediatric and adult patients
Source: Bioengineered. 2021 Oct 21;12(1):8309–24. doi: 10.1080/21655979.2021.1985815 (PMC8806981; doi:10.1080/21655979.2021.1985815)
Supplement: Supplemental Material [file KBIE_A_1985815_SM9367.zip › supplementary/Supplementary Materials.docx]

**Supplementary Materials**

**Supplementary Material 1. Samples details in GSE87473 and GSE126124 datasets.**

**Supplementary Material 2. Clinical characteristics of patients with HC and UC. HC,** Healthy control; UC, Ulcerative colotis.

**Supplementary Material 3. Primers for human and mice qRT-PCR.**

**Supplementary Material 4. A list of DEGs.** DEGs: Differentially expressed genes.

**Supplementary Material 5.** **Enrichment analyses of genes functions in pediatric and adult UC.** (A) Top 20 shared up-regulated genes enriched functions. (B) Top 20 shared down-regulated genes enriched functions. UC, Ulcerative colitis.

**Supplementary Material 6. Butyrate metabolism pathways.** The crucial biochemical pathways (blue arrow) associated with selected genes (red text) are highlighted.
