# Supplementary figures and images for: Evidence for the butyrate metabolism as key pathway improving ulcerative colitis in both pediatric and adult patients
Source: Bioengineered. 2021 Oct 21;12(1):8309–24. doi: 10.1080/21655979.2021.1985815 (PMC8806981; doi:10.1080/21655979.2021.1985815)

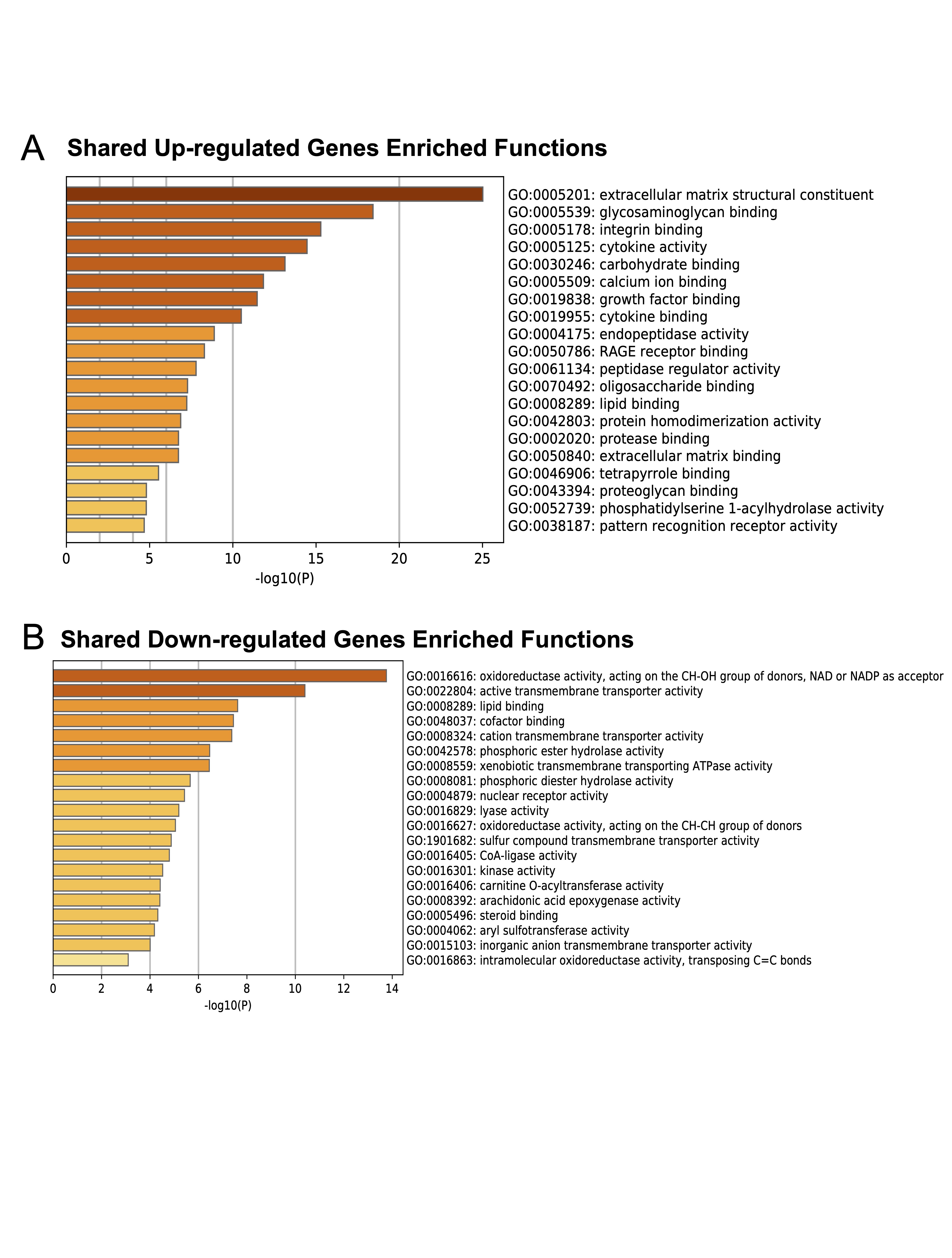

Supplement: Supplemental Material [file KBIE_A_1985815_SM9367.zip › supplementary/Supplementary Material 5.jpg]

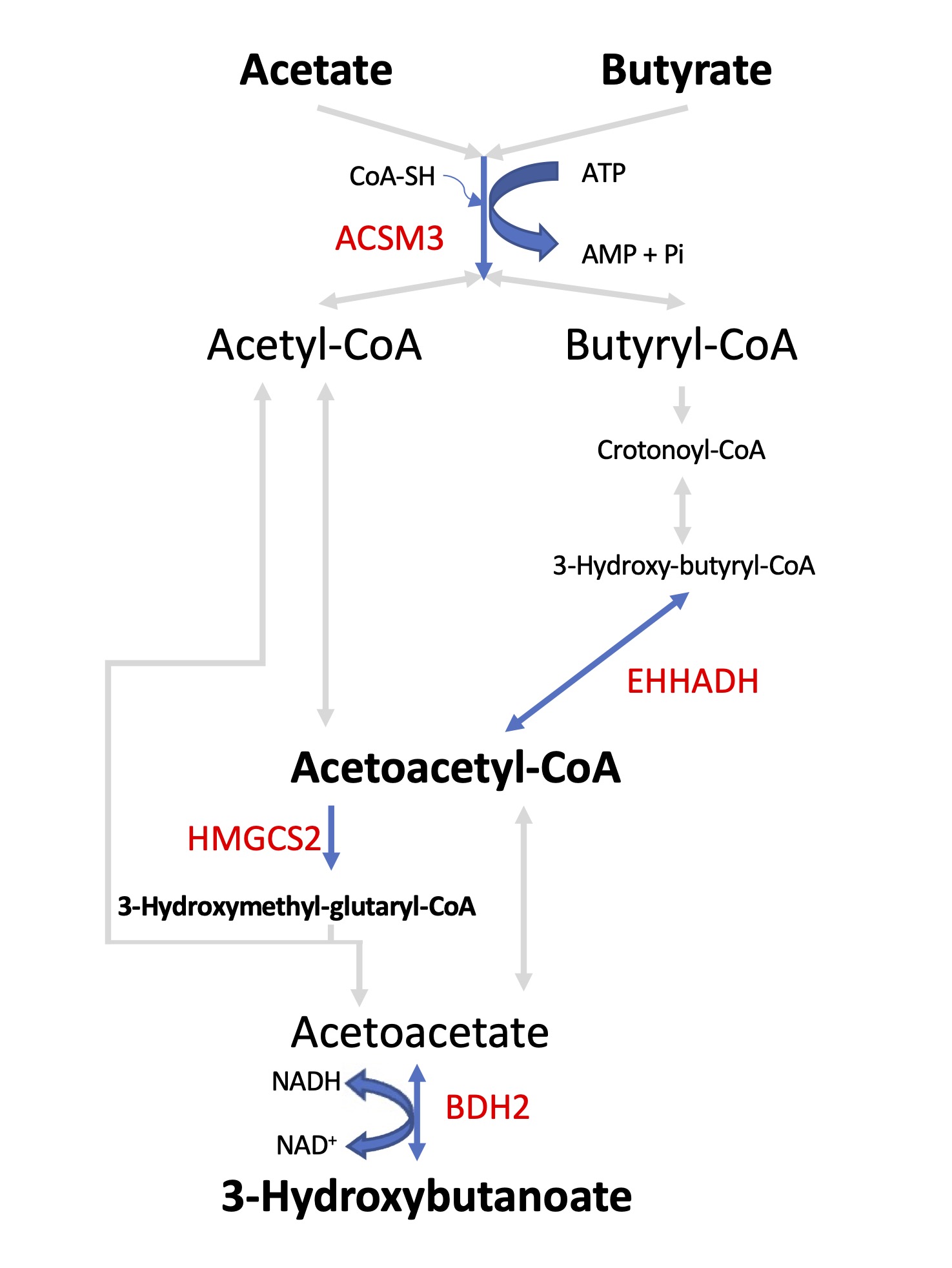

Supplement: Supplemental Material [file KBIE_A_1985815_SM9367.zip › supplementary/Supplementary Material 6.jpg]
